# Estimating bacteria diversity in different organs of nine species of mosquito by next generation sequencing

**DOI:** 10.1186/s12866-018-1266-9

**Published:** 2018-10-04

**Authors:** M V Mancini, C Damiani, A Accoti, M Tallarita, E Nunzi, A Cappelli, J Bozic, R Catanzani, P Rossi, M Valzano, A Serrao, I Ricci, R Spaccapelo, G Favia

**Affiliations:** 1 School of Biosciences and Medical Veterinary, University of Camerino, Via Gentile III da Varano, 62032 Camerino, MC Italy; 2 Department of Experimental Medicine, Functional Genomics Center, University of Perugia, Via Lucio Severi 1, 06132 Perugia, Italy; 3 Present Address: Centre for Virus Research, Level 3 Henry Wellcome Building, 464 Bearsden Road, Glasgow, UK

**Keywords:** Symbionts, Mosquitoes, Metagenomics

## Abstract

**Background:**

Symbiosis in insects is accumulating significant amount of studies: the description of a wide array of mutualistic associations across the evolutionary history of insects suggests that resident microbiota acts as a driving force by affecting several aspects of hosts biology.

Among arthropods, mosquito midgut microbiota has been largely investigated, providing crucial insights on the role and implications of host-symbiont relationships. However, limited amount of studies addressed their efforts on the investigation of microbiota colonizing salivary glands and reproductive tracts, crucial organs for pathogen invasion and vertical transmission of symbiotic microorganisms. Using 16S rRNA gene sequencing-based approach, we analysed the microbiota of gut, salivary glands and reproductive tracts of several mosquito species, representing some of the main vectors of diseases, aiming at describing the dynamics of bacterial communities within the individual.

**Results:**

We identified a shared core microbiota between different mosquito species, although interesting inter- and intra-species differences were detected. Additionally, our results showed deep divergences between genera, underlining microbiota specificity and adaptation to their host.

**Conclusions:**

The comprehensive landscape of the bacterial microbiota components may ultimately provide crucial insights and novel targets for possible application of symbionts in innovative strategies for the control of vector borne diseases, globally named Symbiotic Control (SC), and suggesting that the holobiont of different mosquito species may significantly vary. Moreover, mosquito species are characterized by distinctive microbiota in different organs, likely reflecting different functions and/or adaptation processes.

**Electronic supplementary material:**

The online version of this article (10.1186/s12866-018-1266-9) contains supplementary material, which is available to authorized users.

## Background

Insects represent the most diverse and abundant clade of metazoans, accounting for more than 90% species and dominating a variety of terrestrial habitats. Diverse insect habits are founded on associations with microorganisms, whose diversity reflects the variety of their hosts [1]. These complex associations are passed between generations and can assume mutualism or commensalism features. The most important distinction is whether this microbiota is transient (acquired from the surrounding environment) or it is indigenous to its host, maintaining stable communities and colonizing the gut habitat [2].

Various insects host resident microorganisms able to influence many insect’s key functions, although not primarily. Diverse physiological, metabolic and immune processes of hosts are significantly influenced by their microbiota, whose colonization significantly contributed to their evolutionary success. Coexistence and coevolution may rely on increased microbial production of nutrients valuable to the host and correlated changes in its metabolism, promoting host complementarity to microbial nutrients [3]. An elegant study describes symbiotic bacteria of *Drosophila melanogaster* capable of affecting mating preferences by changing the levels of cuticular hydrocarbons and sex pheromones [4]. Additionally, endosymbionts were found to influence dispersal behaviour, species distribution and overall community composition in spiders [5, 6]. Symbiotic bacteria also impact on body colours of aphids, influencing prey-predator interactions, as well as interactions with other endogenous endosymbionts. Interestingly, insect life cycles are also dependent on microbiome: some mosquito species were reported to rely upon their gut microbiota for molting and developing from larvae to adults [7]. In addition, microbiota contribution on the well-being of the host is also revealed by its ability to promote the resistance of insects to certain natural enemies, including viruses, bacteria, fungi, nematodes, parasites, and in turn, to modulate vector capacity of some insects involved in transmission of infectious diseases. Moreover, symbionts are shown to contribute to insecticide resistance phenomena [8].

In this frame, among insects, mosquitoes represent an important public health challenge in many parts of the globe. *Anopheles* mosquitoes are vectors of human pathogens, including parasites responsible of severe infectious diseases (malaria and lymphatic filariasis) and arboviruses (O’nyong-nyong virus). In sub-Saharan Africa, the main human malaria vectors are members of the *An. gambiae* complex, together with *An. coluzzii*. *An. stephensi*, instead, represents one of the major malaria vector in Asia.

Mosquitoes of the genus *Aedes* are worldwide involved in outbreaks of arboviruses, including Dengue, Chikungunya and Zika viruses [9]. *Aedes* mosquitoes are known to be highly invasive with a great capacity to adapt to contrasting climates and environments. Mosquitoes belonging to the *Culex* genus were found to be involved in the transmission of human and animal diseases, such as West Nile fever, often acting as a bridge vector between birds and humans [10]. Additionally, these genera exhibit different and specific rhythmic behaviours across their life cycle. For instance, the majority of *Anopheles* and *Culex* species are mainly characterized by nocturnal biting behaviour, whereas *Aedes* typically engage biting activities in the daytime. They also differ in the choice of the breeding site: *Anopheles* mostly prefer clear water exposed to sunlight, *Culex* and *Aedes* are instead mostly found in dark water containing organic matter [11].

Previous studies have described midgut microbiota of mosquitoes as a driving force to directly and/or indirectly affects host-pathogen interactions, and ultimately vector capacity, significantly influencing diseases transmission. In *Anopheles* mosquitoes, resident microbiota inhibits the invasion of ookinetes of the malaria parasite *Plasmodium* of the midgut epithelium, reducing infection rate: this effect is mediated by colonizing bacteria, and not caused by their direct interaction with *Plasmodium* [12]. Also, arboviruses transmission undergoes microbiota-mediated modulations within the mosquito, mainly due to its indirect influence on nutrient catabolism, development, and immune responses [7, 13, 14]. This is particularly remarkable with viruses because of their strict dependence on host factors for invasion and replication processes. Furthermore, microbiota can directly interact with arboviruses by inhibiting viral transmission through the secretion of anti-viral compounds [15]. On the other hand, bacteria could also act as pro-factors by increasing vector permissibility to arboviral infections [16, 17].

Microbiota in mosquitoes is often restricted to bacteria associated to the gut. This tissue represents a reservoir of a wide variety of microbial communities, whose characterization is essential and preparatory for a solid understanding of the overall vector biology [18]. Nevertheless, other organs result to be inhabited by microorganisms: some taxa are shared with the intestinal tract, while some others showed preferences for different tissues. Only recently, a comprehensive study addressed the question about the various and diverse composition of the microbiota of midguts, ovaries, and salivary glands of female individuals of *An. gambiae* and *An. coluzzii*, using pyrosequencing analysis of the 16S rRNA gene [19]. Additionally, the analysis of the salivary glands of *An. culicifacies* revealed to host a more complex and various microbiome than that inhabiting the gut [20], underlying diverse bacterial colonization abilities, and in particular the intrinsic environmental divergence offered by organs, despite belonging to the same individual.

We investigated nine species of mosquitoes belonging to the main representative mosquito genera, in terms of geographical diffusion and public health interest. This includes six anopheline species [*An. gambiae* (G3 and Kisumu strains), *An. coluzzii*, *An. arabiensis*, *An. quadriannulatus, An. merus*, *An. stephensi*], two belonging to the *Aedes* genus (*Ae. aegypti*, *Ae. albopictus*) and *Cx. quinquefasciatus*. Herein, data of an extensive study aimed to characterize bacterial microbiota associated to gut, salivary glands and reproductive organs are reported. The importance of describing the microbiota of these organs lies in possibility to develop innovative paratransgenic approaches for vector control. The selection of suitable symbiont candidates primarily requires a specific tissue tropism in the gut, the salivary glands and the reproductive organs [21], since i) the transmission cycle of many pathogens starts in the intestinal tract and culminates with the invasion of the salivary glands from where they are transmitted to a new host and ii) the colonization of the reproductive organs allows the vertical transmission of bacteria to the offspring, ensuring their persistence within a population.

## Results

### Overall distribution of bacteria within different organs

**Fig. 1:**
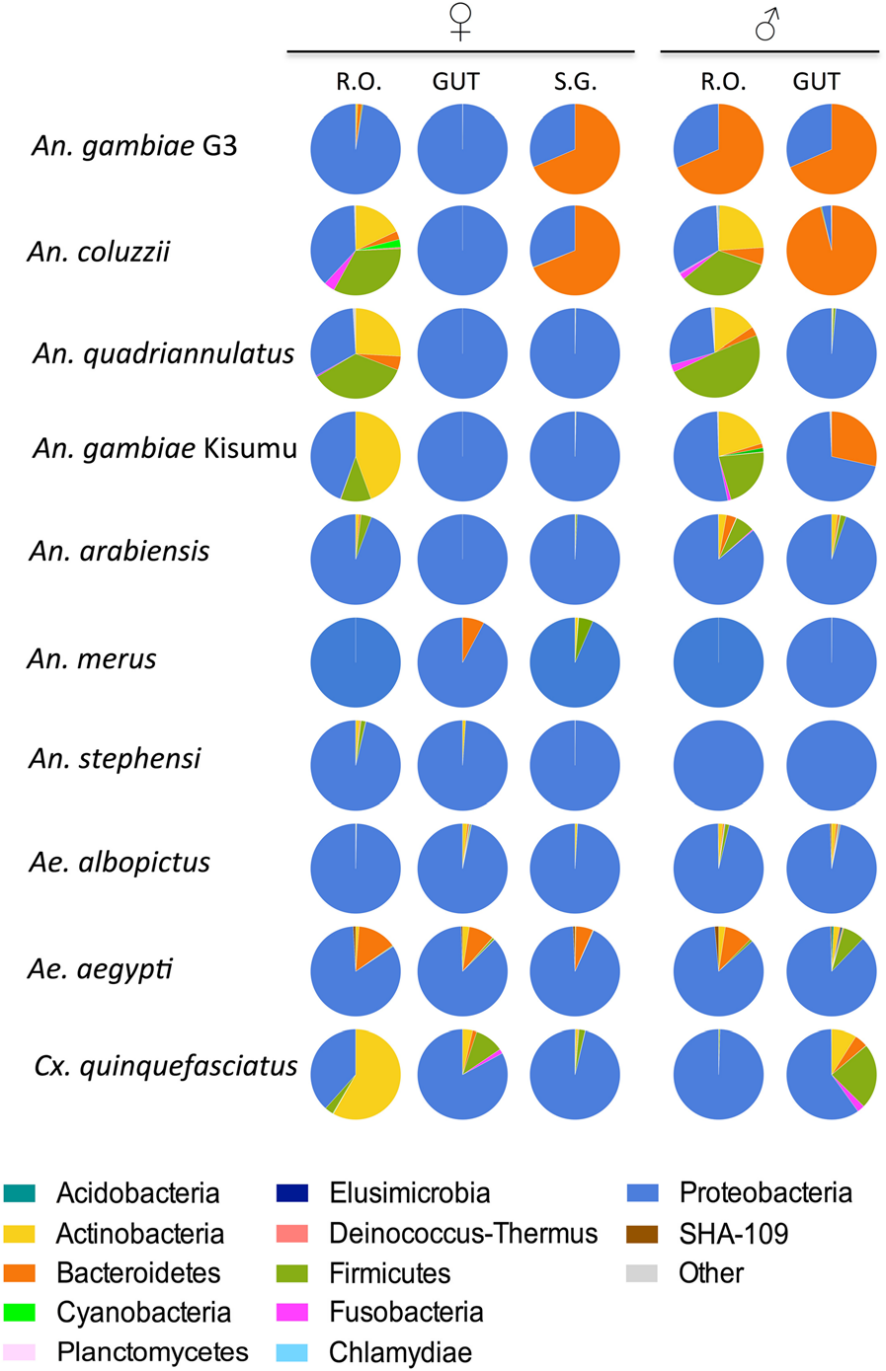
Phylum level composition (% of OTUs) in different organs of nine mosquito species. All OTUs are represented, except the unassigned (0,1%). R.O.: reproductive organs; S.G.: salivary glands; ♀: females; ♂: males

A total of 951.930 reads were generated after removal of short reads (< 250 bp), chimeras and the discard of spurious OTUs [22] from all tissues analyzed. The number of reads varied among samples (minimum = 2228, maximum = 62,851) (Table 1), therefore the number of sequences for each sample was rarefied to the minimal readings of 2228. The sequences were clustered in 924 bacterial OTUs (Table 1). Analysis of the rarefaction curves indicated an adequate sampling quality; only in a few cases, the curves did not reach the saturation to an asymptote, indicating the presence of additional rare bacteria taxa or further spurious OTUs (Additional file 1: Figure S1). Furthermore, the number of observed species estimator was used to evaluate bacteria richness between the mosquito species/strains (Additional file 2: Figure S2). Interestingly, irregular diversity in terms of bacterial abundance is highlighted when indices are compared. Bacterial richness of reproductive organs is higher than that found in the other tissues (Table 1). In most samples, salivary glands harbour a more diverse microbiota than the gut. In particular, *Ae. aegypti* harbours the highest number of bacteria species compared to others, followed by *An. coluzzii* and *An. quadriannulatus*. *An. gambiae* G3 and *An. stephensi* contain the lowest number of bacteria species, explained by the long adaptation in laboratory conditions (Additional file 2: Figure S2). In total, mosquito tissues (guts, reproductive organs and salivary glands) host a flora consisting of bacterial OTUs belonging to 12 phyla, 74 families and 121 genera. Among these, 4 phyla represent more than 99% of the total microbiota: Proteobacteria*,* Bacteroidetes, Firmicutes and Actinobacteria (Fig. 1, Additional file 3: Table S1). The OTUs assigned to other not abundant phyla (< 1%) included: Fusobacteria, Acidobacteria, Cyanobacteria, Chlamydiae, Deinococcus-Thermus, Elusimicrobia, Planctomycetes and SHA-109. Some bacteria could not be assigned to any taxa (0.1%). The phylum Proteobacteria is dominant in the majority of tissues, showing to be the main constituent of a shared and conserved microbiota core (Fig. 1). Striking examples of Proteobacteria abundance (> 60%) are found in the following organs: i) guts of females; ii) guts of males, except for *An. gambiae* G3 and *An. coluzzii*; iii) salivary glands of all species apart from *An. gambiae* G3 and *An. coluzzii*; iv) the reproductive organs of both males and females of *An. arabiensis, An. merus, An. stephensi, Ae. albopictus* and *Ae. aegypti,* the ovaries of *An. gambiae* G3 and organs of *Cx. quinquefasciatus* males*.* However, Bacteroidetes (> 68%) are present in male guts and salivary glands of both *An. gambiae* G3 and *An. coluzzii*, and in the reproductive organs of *An. gambiae* G3 males. The guts of *An. gambiae* Kisumu males harbour more than 28% of Bacteroidetes, which also encompasses 10% of taxa in *Ae. aegypti* organs. Firmicutes and Actinobacteria strongly colonize the reproductive organs of both males and females of *An. coluzzii, An. quadriannulatus* and *An. gambiae* Kisumu, whereas they are found only in ovaries of *Cx. quinquefasciatus.*

**Table 1:** Bacterial diversity and richness in salivary glands, guts and reproductive organs of mosquito species

Mosquitoes	Sex	Organ	N. reads	Observed OTUs	Shannon	Simpson
*An. gambiae* G3	♀	s.g.	10,775	66	2038	0,752
		guts	18,555	222	2364	0,691
		r.o.	22,418	326	2719	0,739
	♂	guts	13,918	78	2013	0,746
		r.o.	13,918	78	1947	0,699
*An. coluzzii*	♀	s.g.	10,959	116	2229	0,766
		guts	17,160	157	1910	0,595
		r.o.	62,851	467	4930	0,979
	♂	guts	10,105	57	1561	0,650
		r.o.	62,732	448	4670	0,971
*An. quadriannulatus*	♀	s.g.	17,056	296	2903	0,797
		guts	22,176	236	3267	0,853
		r.o.	53,254	427	4813	0,974
	♂	guts	18,000	137	1935	0,596
		r.o.	13,540	323	4373	0,954
*An. gambiae* Kisumu	♀	s.g.	22,653	343	2772	0,744
		guts	15,551	239	3109	0,797
		r.o.	10,160	181	3354	0,910
	♂	guts	13,774	209	2693	0,843
		r.o.	59,029	472	4836	0,977
*An. arabiensis*	♀	s.g.	28,828	337	2865	0,831
		guts	20,354	192	2105	0,631
		r.o.	10,021	294	3605	0,907
	♂	guts	26,666	465	3675	0,898
		r.o.	13,884	363	4567	0,968
*An. merus*	♀	s.g.	3553	175	3286	0,896
		guts	24,600	218	2530	0,754
		r.o.	2228	11	1211	0,619
	♂	guts	27,009	258	2941	0,853
		r.o.	5548	10	0,980	0,564
*An. stephensi*	♀	s.g.	23,533	196	2416	0,720
		guts	18,922	233	2103	0,605
		r.o.	15,628	302	3898	0,953
	♂	gut	16,619	157	1905	0,588
		r.o.	15,070	209	2647	0,782
*Ae. albopictus*	♀	s.g.	12,976	218	2395	0,687
		guts	17,025	387	3791	0,921
		r.o.	16,078	114	2607	0,866
	♂	guts	22,371	382	3759	0,913
		r.o.	8960	237	3372	0,894
*Ae. aegypti*	♀	s.g.	19,189	385	5019	0,975
		guts	20,603	427	4899	0,978
		r.o.	26,064	410	5025	0,973
	♂	guts	3167	184	3781	0,938
		r.o.	5007	251	4382	0,965
*Cx. quinquefasciatus*	♀	s.g.	18,083	290	3304	0,885
		guts	4268	146	2863	0,744
		r.o.	2945	97	2777	0,840
	♂	guts	16,763	250	3744	0,888
		r.o.	17,384	79	2835	0,874

♀: females; ♂: males; s.g.: salivary glands; r.o.: reproductive organs

**Fig. 2:**
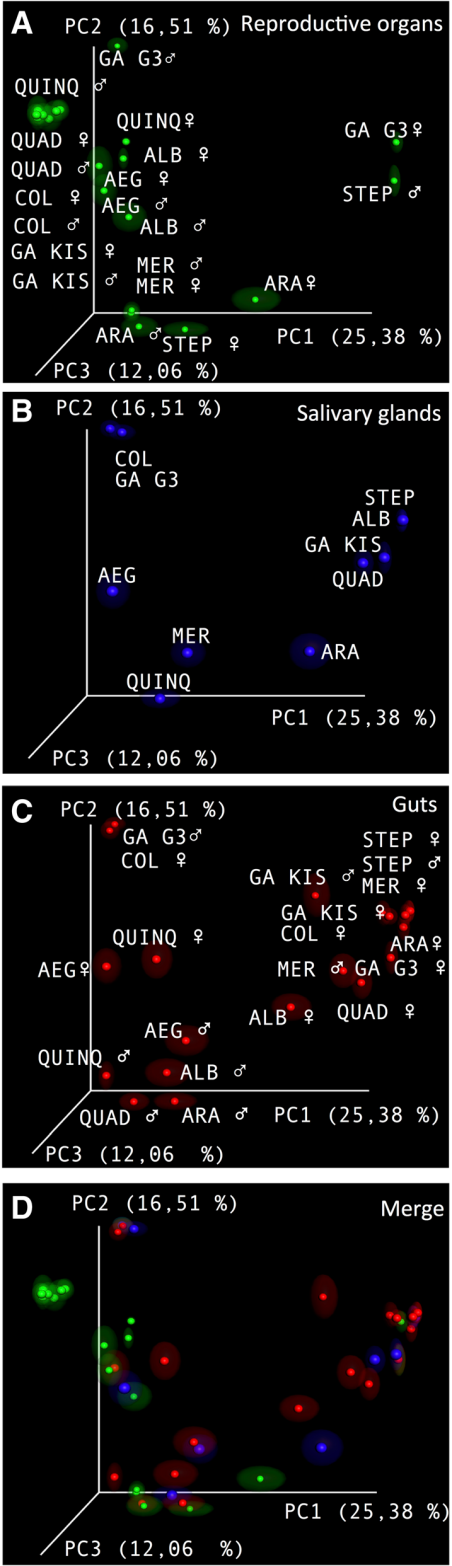
Principal Coordinates Analysis (PCoA) plots of samples colored according to different organs. **a** Mosquitoes reproductive organs from females (♀) and males (♂). **b** Salivary glands from female mosquito species. **c** Mosquito guts from females (♀) and males (♂). **d** Merge figure from panel **a**, **b** and **c**. GA G3: *An. gambiae* G3; COL: *An. coluzzii*; QUAD: *An. quadriannulatus*; GA KIS: *An. gambiae* Kisumu; ARA: *An. arabiensis*; MER: *An. merus*; STEP: *An. stephensi*; ALB: *Ae. albopictus*; AEG: *Ae. aegypti*; QUINQ: *Cx. quinquefasciatus*

Consequently, a principal coordinate analysis (PCoA) plot was generated, revealing that the reproductive organs of *An. stephensi* males and *An. gambiae* G3 females are divergent from other samples (Fig. 2a). This sharp difference is mostly caused by abundant presence of *Serratia*. Moreover, the reproductive organs of *An. gambiae* G3 males show a unique cluster due to the large presence of *Elizabethkingia*. The relevant presence of members of the *Elizabethkingia* genus also defines a cluster in the salivary glands of *An. gambiae* G3 and *An. coluzzii* (Fig. 2 b); an additional group is composed by guts of *An. gambiae* G3 males and *An. coluzzii* females (Fig. 2c). The rest of the samples tends to cluster together (Fig. 2a-b-c ). A global representation is described in the merged picture (Fig. 2d).

### Microbiota composition of reproductive organs

**Fig. 3:**
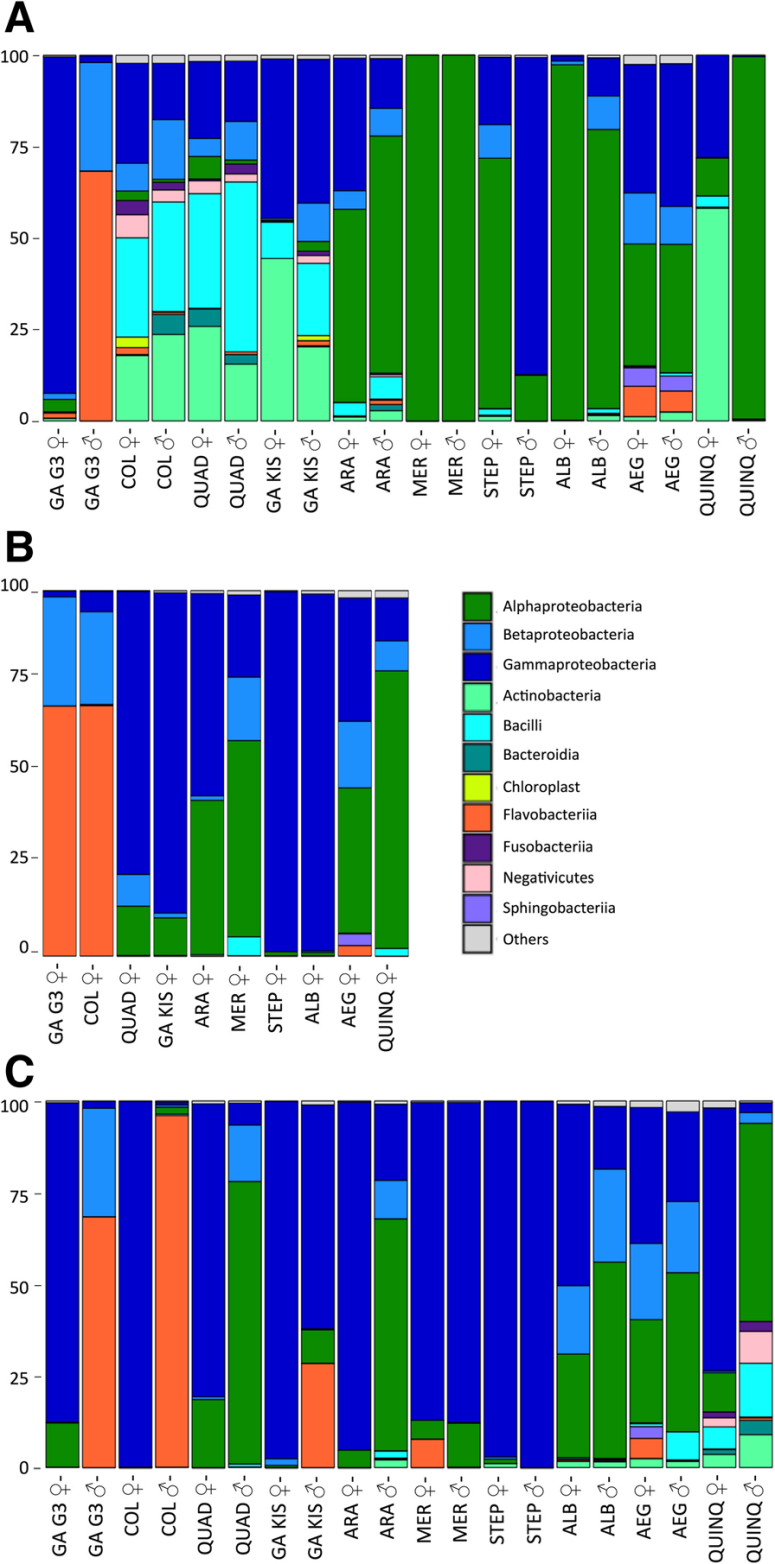
Class level composition (% of OTUs) in different organs of nine mosquito species. Only OTUs representing > 1% of the total reads are represented here. The class color code is given for (**a**), (**b**) and (**c**). GA G3: *An. gambiae* G3; COL: *An. coluzzii*; QUAD: *An. quadriannulatus*; GA KIS: *An. gambiae* Kisumu; ARA: *An. arabiensis*; MER: *An. merus*; STEP: *An. stephensi*; ALB: *Ae. albopictus*; AEG: *Ae. aegypti*; QUINQ: *Cx. quinquefasciatus*; ♀: females; ♂: males

When classified by classes, although irregularly abundant, Alphaproteobacteria are present in most samples, almost representing the total microbiota of *Ae. albopictus* (♀: 97%; ♂: 73%) and *Cx. quinquefasciatus* (♂: 99%); whereas in *An. gambiae* G3, *An. coluzzii* males, *An. gambiae* Kisumu females, it accounts for less than 1% of the OTUs. Lower abundance is detected in *An*. *arabiensis* (♀: 33%, ♂: 42%), *An. stephensi* (♀: 48%), *Ae. aegypti* (♀ and ♂ 32%), *Cx. quinquefasciatus* (♀: 13%) and between 1 and 6% in the remaining samples (Fig. 3a).

Gammaproteobacteria and Betaproteobacteria is abundant in most samples, except for Gammaproteobacteria in *Cx. quinquefasciatus* males (< 1%) and for Betaproteobacteria in *Cx. quinquefasciatus* males and females, *An. stephensi* and *An. gambiae* Kisumu females (Fig. 3a).

On the contrary, reproductive organs of the species of the *An. gambiae* complex harbour a dissimilar microbiota: the class of Bacilli is predominant, followed by Gammaproteobacteria, Actinobacteria, Betaproteobacteria and Fusobacteria. However, *An. gambiae* G3 represents an exception, where Gammaproteobacteria (85%) in females and Flavobacteria (74%) in males dominate the overall microbiota (Fig. 3a).

**Fig. 4:**
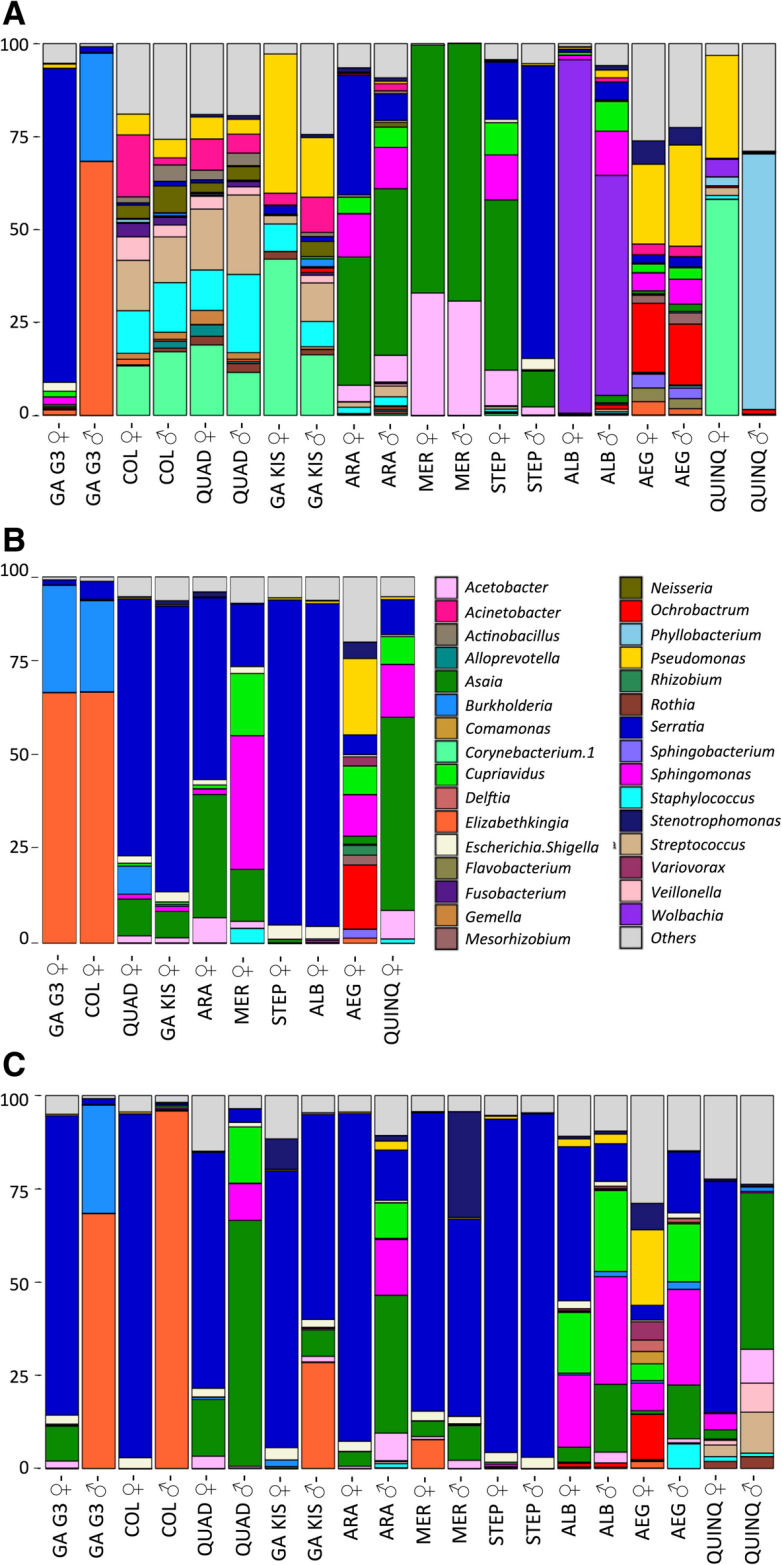
Genus level composition (% of OTUs) in different organs of nine mosquito species. Only OTUs representing > 1% of the total reads are represented here. The genus color code is given for (**a**), (**b**) and (**c**). GA G3: *An. gambiae* G3; COL: *An. coluzzii*; QUAD: *An. quadriannulatus*; GA KIS: *An. gambiae* Kisumu; ARA: *An. arabiensis*; MER: *An. merus*; STEP: *An. stephensi*; ALB: *Ae. albopictus*; AEG: *Ae. aegypti*; QUINQ: *Cx. quinquefasciatus*; ♀: females; ♂: males

Despite a few exceptions, the analysis of taxa at genus level showed the persistence of a shared core of bacteria. *An. gambiae* G3 ovaries are mainly colonized by *Serratia* (78%) followed by *Escherichia-Shigella* (0.5%), *Sphingomonas* (0.2%), *Cupriavidus* (0.2%) and *Elizabethkingia* (0.2%). The latter, on the contrary, represents the dominant taxon (73%) in male organs, co-habiting with *Burkholderia* (18%) and, in much lower amount, with *Serratia* (0.3%). Similarly, in *An. stephensi*, *Serratia* predominantly inhabits male reproductive organs, while in ovaries it is exceeded by *Asaia,* whose presence in males is reduced. Finally, *Cx. quinquefasciatus* offered a divergent scenario: male reproductive organs are mainly colonized by *Phyllobacterium* (91%), although its contribution in ovaries microbiota was minimal (0.8%) (Fig. 4a). Differently, the ovaries of *Cx. quinquefasciutus* host *Serratia*, and several genera of the families Rhodobacteriaceae and Rhizobiaceae (Additional file 4: Figure S3). As expected, *Wolbachia* colonization in *Ae. albopictus* shows a noticeable predominance in ovaries (94%); in male reproductive organs, it coexists with *Sphingomonas, Cupriavidus and Serratia*.

Finally, the reproductive organs of *Ae. aegypti* show an interestingly high richness of taxa: *Ochrobactrum, Pseudomonas, Sphingomonas, Stenothropomonas, Acinetobacter* and *Elizabethkinghia* are the most abundant genera detected in female and male individuals (Fig. 4a).

### Microbiota composition of salivary glands

Female salivary glands are also collected and analysed. Gammaproteobacteria represents the most prevalent class in anophelines (*An. quadriannulatus, An. gambiae* Kisumu, *An. arabiensis*, and *An. stephensi)* and in *Ae. albopictus*, and, although less abundant, in *An. merus*, in *Ae. aegypti* and in *Cx. quinquefasciatus.* Alphaproteobacteria, followed by Betaproteobacteria, constitutes the remaining portion (Fig. 3b).

*Serratia* is present in each sample of salivary glands, and together with *Escherichia-Shigella*, *Pantoea, Acetobacter, Sphingomonas, Burkholderia* and *Cupriavidus* is part of a shared core of taxa. *An. quadriannulatus, An. gambiae* Kisumu, *An. arabiensis* are prevalently colonized by *Serratia* and *Asaia*. *An. merus* hosts *Sphingomonas* (34%), *Asaia* (14%) and in lower proportion *Serratia* and *Cupriavidus*. A completely divergent pattern is visible in *An. gambiae* G3 and *An. coluzzii* mosquitoes, where 69% of OTUs are addressed to the *Elizabethkingi*a sp. genus, whose abundance reaches almost undetectable levels in the salivary glands of other species (apart from 1.4% in *Ae. aegypti*). In addition, salivary glands of *An. gambiae* G3 and *An. coluzzii* are inhabited by Betaproteobacteria (genus *Burkholderia*, > 25%), whose colonization is found in lower amount (7.6%) in *An. quadriannulatus* samples.

*Ae. albopictus* is mainly colonized by *Serratia* (88%) like *An. stephensi*. Similarly to other organs, a conserved core of communities is found in salivary glands, consisting of *Asaia, Pseudomonas, Sphingomonas, Cupriavidus, Serratia* and *Escherichia-Shigella.* Moreover, other taxa are identified, including *Acetobacter* and *Ochrobactrum* (Alphaproteobacteria) and *Elizabethkingia* (Bacteroidetes) (Fig. 4b).

### Microbiota composition of guts

The most diffused bacteria classes in anopheline is Gammaproteobacteria, mainly represented by *Serratia*, apart from *An. gambiae* G3 and *An. coluzzii* males, where Flavobacteria (represented by *Elizabethkingia*) reaches 78% and 95% of OTUs, respectively (Fig. 3c, Fig. 4c). *Aedes* microbiota is characterized by a higher diversity in guts, showing Alpha, Beta and Gammaproteobacteria equally colonizing the intestinal tissues (Fig. 3c). Detected genera are also *Sphingomonas*, *Asaia*, *Cupriavidus*, *Escherichia-Shigella*, *Pseudomonas* and *Serratia* (Fig. 4c)*.* Finally, *Cx. quinquefasciatus* shows a unique composition, disclosing a variety of genera; however, Alphaproteobacteria (*Asaia*) are dominant in females, while Gammaproteobacteria (*Yersinia*) largely colonize male guts (Fig. 3c, Fig. 4c).

## Discussion

Through a 16S rRNA gene sequencing-based approach, a description of microbiota diversity hosted in salivary glands, reproductive tracts and guts of several mosquito species is provided. High richness and diversity of microbes associated with laboratory reared individuals was detected, suggesting the persistence of a well-established and conserved core of bacteria. The evidence of a shared microbiota core is consistent with other studies reporting similar data and identifying a large conserved group of bacteria colonizing different mosquito species and their tissues, also in standardized rearing environments. [19, 23, 24].

We are aware that bacterial DNA contamination in extraction kits and laboratory reagents can significantly affect the results of microbiota studies, particularly when samples contain low microbial biomass and when highly sensitive techniques are used. Previous studies described the list of contaminants detected in sequenced negative blank controls derived from reagents contamination, users and extraction kits [25]. The proposed list of contaminants has been compared to the obtained data. Only a few candidates, like *Mesorhizobium*, *Phyllobacterium*, *Rhizobium*, *Comamonas*, *Delftia*, *Variovorax*, *Escherichia-Shigella* were detected at very low percentages in our samples. It has been also reported that *Corynebacterium*, *Propionibacterium* and *Streptococcus* are common human skin-associated organisms. Only *Corynebacterium* and *Streptococcus* were detected in very low percentage (< 1%) in some samples. Overall, the incidence of bacteria contaminations in our data appears minimal; moreover, the taxa described above have been previously presented in metagenomics studies of insects, and for this reason they have been included in the data analysis.

Previous studies proved that commensal bacteria are mainly acquired from the environment or transmitted directly between hosts to their offspring. Often, these contributions are correlated, meaning that bacteria with complete transmission among hosts will evolve specialization for a particular niche [26–28]. Although bacterial composition is selected by standardized diet and rearing conditions, differences have been disclosed and data are able to provide crucial insights for a robust characterization and comparison between species. Additionally, despite belonging to the same individual, tissues specifically harboured diverse bacteria, whose distribution reflects tissue-bacteria adaptations. Within the same individual, microbiota of reproductive organs was more diverse than that of the gut and salivary glands. Salivary glands, mostly in anophelines, showed higher diversity indices when compared to the guts, similarly to what reported in *An. culicifacies* [20]. A leading example of this characteristic adaptation is *Elizabethkingia*: it largely colonizes guts and reproductive organs of *An*. *gambiae* G3 males, while in *An. coluzzii*, it is prevalent in guts of males and salivary glands of females. Its association with guts of *An. gambiae* was already reported in mosquitoes reared in standard insectary conditions, suggesting the establishment of an own thriving niche, and possibly explained with the ability of Bacteroidetes to degrade sugar [29]. Similarly, *Serratia*, although presenting variable abundances in each sample, is found to be dominant in the gut of female anophelines, being consistent with recently reported data [19, 30]. For instance, in *An. stephensi*, *Serratia* represents more than 90% of detected taxa in all organs, except for female ovaries where its abundance is reduced.

Notably, *Asaia* occurrence is in line with its role among symbiotic bacterial communities: it rarely showed, in fact, dominant behaviour, like *Serratia* or *Elizabethkingia*, but its persistence is found in guts and salivary glands of all mosquito genera and its relevant occurrence in reproductive organs of males and females of *An. stephensi* and *An. arabiensis* provides evidences of its widespread colonization ability.

Bacteria such as *Asaia*, *Pantoea*, *Pseudomonas*, *Entrobacter*, *Serratia* are gaining momentum as promising candidates for paratransgenic modifications for vector control strategies. Our findings confirm their abundance in key tissues of various mosquito species, corroborating and widening their role as broad-spectrum tools against mosquito borne diseases. *Cupriavidus* and *Ochrobactrum* could be also included in this bacterial arsenal: their association with mosquitoes was never reported before, but their recent engineering for biomedical purposes could be also translated in the field of mosquito control [31, 32]. In addition, the presence of some bacteria as *Serratia* and *Wolbachia* in different mosquito species are well-known to provide protective effects from pathogens infections, as reported in several studies [33, 34].

In any case, before any field application of bacterial strains, a detailed characterization and analysis of the potential risks for human and animal health is an imperative.

## Conclusions

Our study describes the composition of microbial communities harboured in different tissues of nine mosquito species, transmitting devastating human diseases. The results highlight the importance of a comprehensive understanding of organisms, intended as holobionts, and thus composed by innumerable interacting communities, whose impact is increasingly apparent. The identification of interesting inter- and intra-species differences, coexisting with a shared core microbiota, suggest a singular adaptation and a tissue-specific tropism. The contextual findings of a deep degree of divergences between genera, underlines microbiota specificity and its adaptation to the host. Moving forward, our data lay the basis for the design of effective vector control strategies, relying on the use of mosquito-associated bacteria.

## Methods

### Mosquito strains

The following laboratory strains were used:

*An. gambiae* G3 (MR4, MRA-112) established in Perugia insectary in 2013; *An. gambiae* Kisumu (MR4, MRA-762) established in Perugia insectary in 2013; *An. coluzzii* (MR4, MRA-860) established in Perugia insectary in 2013 (it is worth to mention that we analysed two strains of *An. gambiae*, due to the high polymorphism of G3, comparing whether the genetic background could influence the microbiota composition); *An. arabiensis* (MR4, MRA-339) established in Perugia insectary in 2013; *An. merus* (MR4, MRA1156) established in Perugia insectary in 2013; *An. quadriannulatus* (MR4, MRA-761) established in Perugia insectary in 2013; *An. stephensi* (SD-500) established in Perugia insectary in 2011; *Ae. albopictus* (MR4, MRA-804) established in Perugia insectary in 2013; *Ae. aegypti* (New Orleans, LA 2011) established in Perugia insectary in 2013; *Cx. quinquefasciatus* (collected Hawaii U.S.A., 2008 and provided by Rutgers University) established in Camerino insectary in 2011.

### Mosquito rearing

Analyzed mosquitoes were all cyclic laboratory-strain colonies. Mosquitoes were reared with a 12 h day/night cycle at 27 °C and relative humidity of 70%. *Anopheles*, *Aedes* and *Culex* larvae were reared in deionized water with 0.3 g/liter of artificial sea salts and fed daily with a diet composed by a slurry of 2:2:1 bovine liver powder, tuna meal and Vanderzant vitamin mix [35]. Adults were fed ab libitum on 10% sucrose.

### Tissues collection and DNA extraction

Prior dissecting, mosquitoes were surface sterilized in 70% ethanol for 5 min and rinsed twice in sterile PBS. Foreguts and midguts, salivary glands lobes and reproductive organs, consisting on testes and male accessory glands (MAGs) for males, and ovaries for females, were dissected with special care to reduce contaminations: needles and dissecting slides were sterilized and treated with 70% ethanol between each sample, while storing vials were UV treated before use. Pooled guts (10 whole guts), reproductive organs (10 sets) and salivary glands (20 pairs) were homogenized with sterile 0.5-mm wide glass beads (Bertin) for 30s at 6800 rpm in the automatic tissue homogenizer (Precellys 24, Bertin). Genomic DNA was extracted using a JetFlex Genomic DNA Purification kit (Genomed) according to the manufacturer’s instructions.

### Amplicon and library preparation

Bacterial 16S V4 regions rDNA were amplified using bacteria/archaeal degenerate primers 515F/806R [36]. Amplification and library preparation were performed as reported previously [37].

### Bioinformatic analysis

Quality control of raw data was done using FastQC, then adapters sequences and low quality scores (< 20) were trimmed by Trimmomatic software package [38]. Cleaned sequenced paired-end reads were merged to reconstruct original full-length 16S amplicons with PEAR software [39]. All amplicons with sequence similarity higher than 97% were chosen as input for making the taxonomy annotation and building the OTU table by following the Open Reference approach. The obtained sequences were searched for matching in the SILVA taxonomy database (v123) using similarity-searching algorithm [40]. The microbial communities of each sample were built from the taxonomy assignments. The alpha-diversity (of each sample) was investigated by means of three different indexes: Shannon and Simpson observed OTUs, in order to quantify the number and distribution of taxa in each sample [41–43]. The dissimilarity analysis between samples measuring population composition (beta-diversity) has been evaluated using QIIME weighted Unifrac distances between samples at a sub-sampling depth of 100 sequences per sample. Thus, jackknifed principal coordinates have been calculated on rarefied results to compress dimensionality into two- and three-dimensional principal coordinate analysis plots. The rarefied results have been used also for computing alpha diversity using observed species, Shannon and phylogenetic diversity (PD) metrics. Data analysis was done using Qiime and the R-software (http://qiime.org, http://www.r-project.org/).

## Additional files

Additional file 1: **Figure S1.** Alpha diversity. Box plots of bacterial species richness associated with the nine mosquito species. The box plots indicate median (middle line), upper and lower quartiles (box top and bottom), minimum and maximum (whiskers). (JPG 3163 kb)

Additional file 2: **Figure S2.** Rarefaction curves. Rarefaction curves calculated for each sample based on the OTU computations, reflect different diversities in different samples and help to estimate whether bacterial communities were sampled properly, i.e. enough sequence reads per sample where collected. Rarefaction curves are expected to reach a plateau if sampling has been exhaustive. ♀: females; ♂: males. (JPG 1442 kb)

Additional file 3: **Table S1.** Percentage of phyla OTUs in the reproductive organs (A) salivary glands (B) and guts (C). (DOCX 47 kb)

Additional file 4: **Figure S3.** Family level composition (% of OTUs) in different organs of nine mosquito species. Only OTUs representing > 1% of the total reads are represented here. The family color code is given for (A), (B) and (C). GA G3: *An. gambiae* G3; COL: *An. coluzzii*; QUAD: *An. quadriannulatus*; GA KIS: *An. gambiae* Kisumu; ARA: *An. arabiensis*; MER: *An. merus*; STEP: *An. stephensi*; ALB: *Ae. albopictus*; AEG: *Ae. aegypti*; QUINQ: *Cx. quinquefasciatus*; ♀: females; ♂: males. (JPG 3132 kb)

## Abbreviations

BLAST: basic local alignment search tool
MAGs: male accessory glands
MR4: The Malaria Research and Reference Reagent Resource Centre
NCBI: National Centre for Biotechnology Information
OTU: operational taxonomic unit
PBS: phosphate buffer saline solution
PCoA: principal coordinate analysis
PD: phylogenetic diversity
PEAR: paired-end read merger
QIIME: quantitative insights into microbial ecology
rRNA: ribosomal RNA
SC: Symbiotic Control
