# Supplementary figures and images for: Estimating bacteria diversity in different organs of nine species of mosquito by next generation sequencing
Source: BMC Microbiol. 2018 Oct 4;18:126. doi: 10.1186/s12866-018-1266-9 (PMC6172810; doi:10.1186/s12866-018-1266-9)

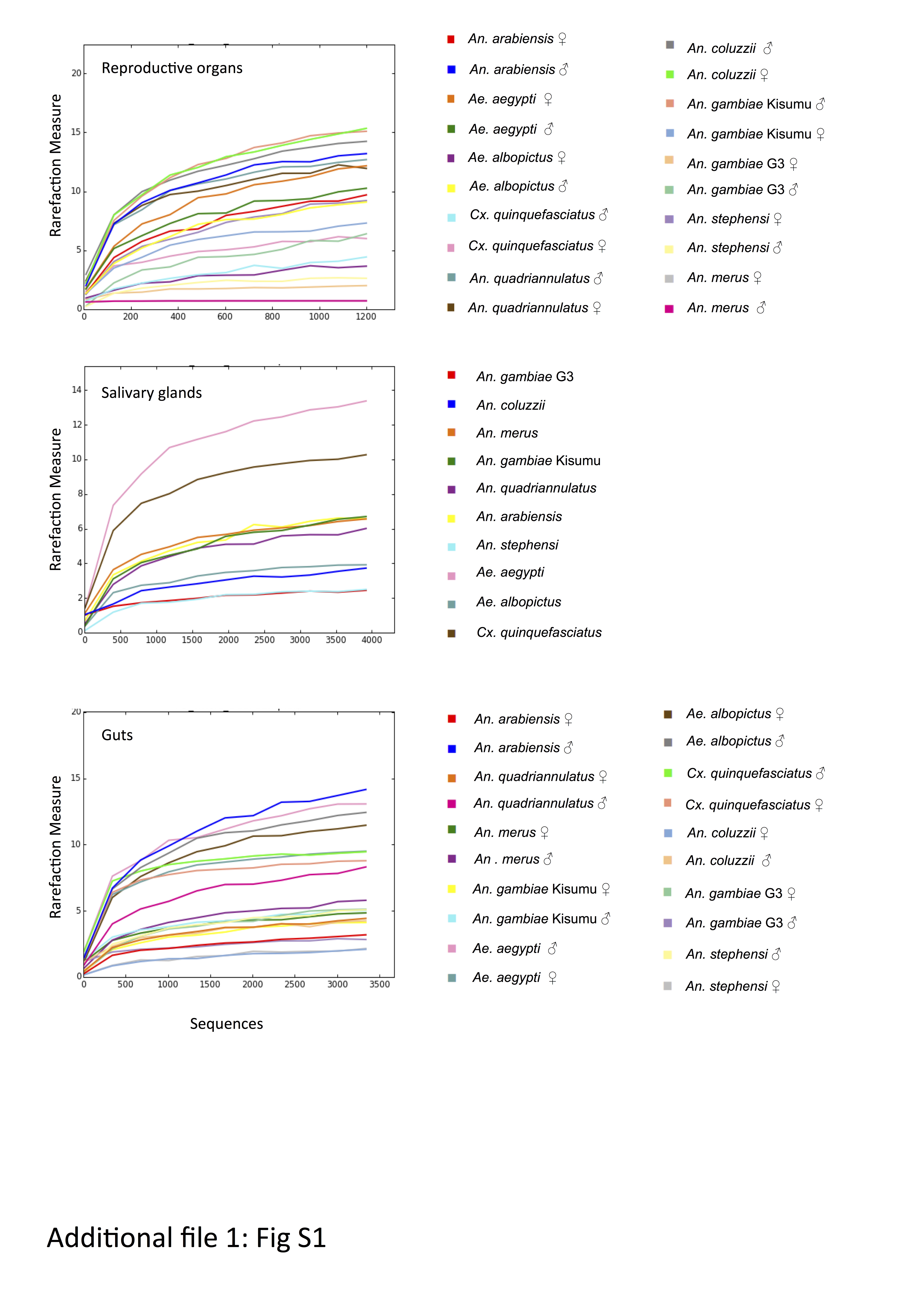

Supplement: Supplementary file 1 — Figure S1. Alpha diversity. Box plots of bacterial species richness associated with the nine mosquito species. The box plots indicate median (middle line), upper and lower quartiles (box top and bottom), minimum and maximum (whiskers). (JPG 3163 kb) [file 12866_2018_1266_MOESM1_ESM.jpg]

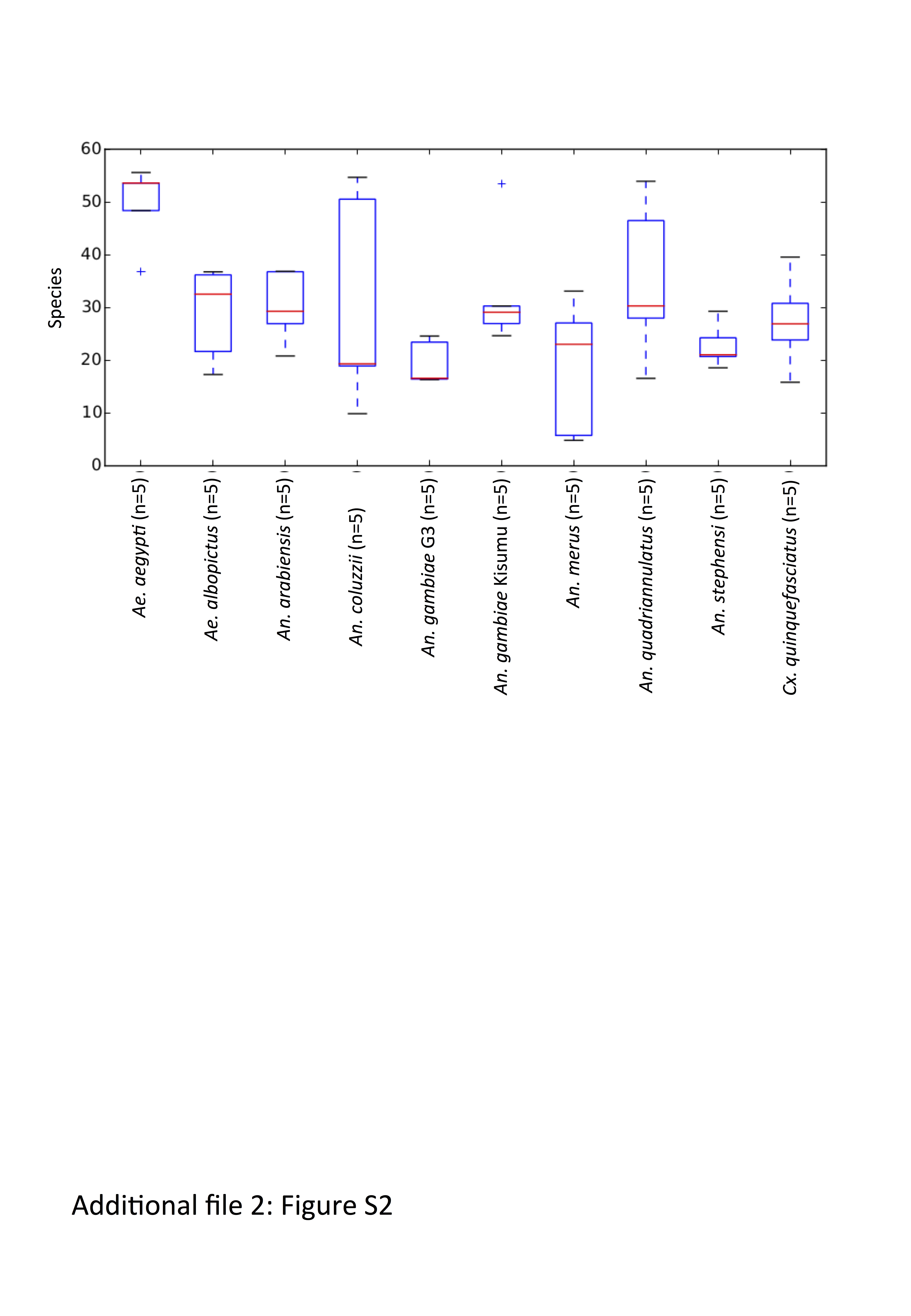

Supplement: Supplementary file 2 — Figure S2. Rarefaction curves. Rarefaction curves calculated for each sample based on the OTU computations, reflect different diversities in different samples and help to estimate whether bacterial communities were sampled properly, i.e. enough sequence reads per sample where collected. Rarefaction curves are expected to reach a plateau if sampling has been exhaustive. ♀: females; ♂: males. (JPG 1442 kb) [file 12866_2018_1266_MOESM2_ESM.jpg]

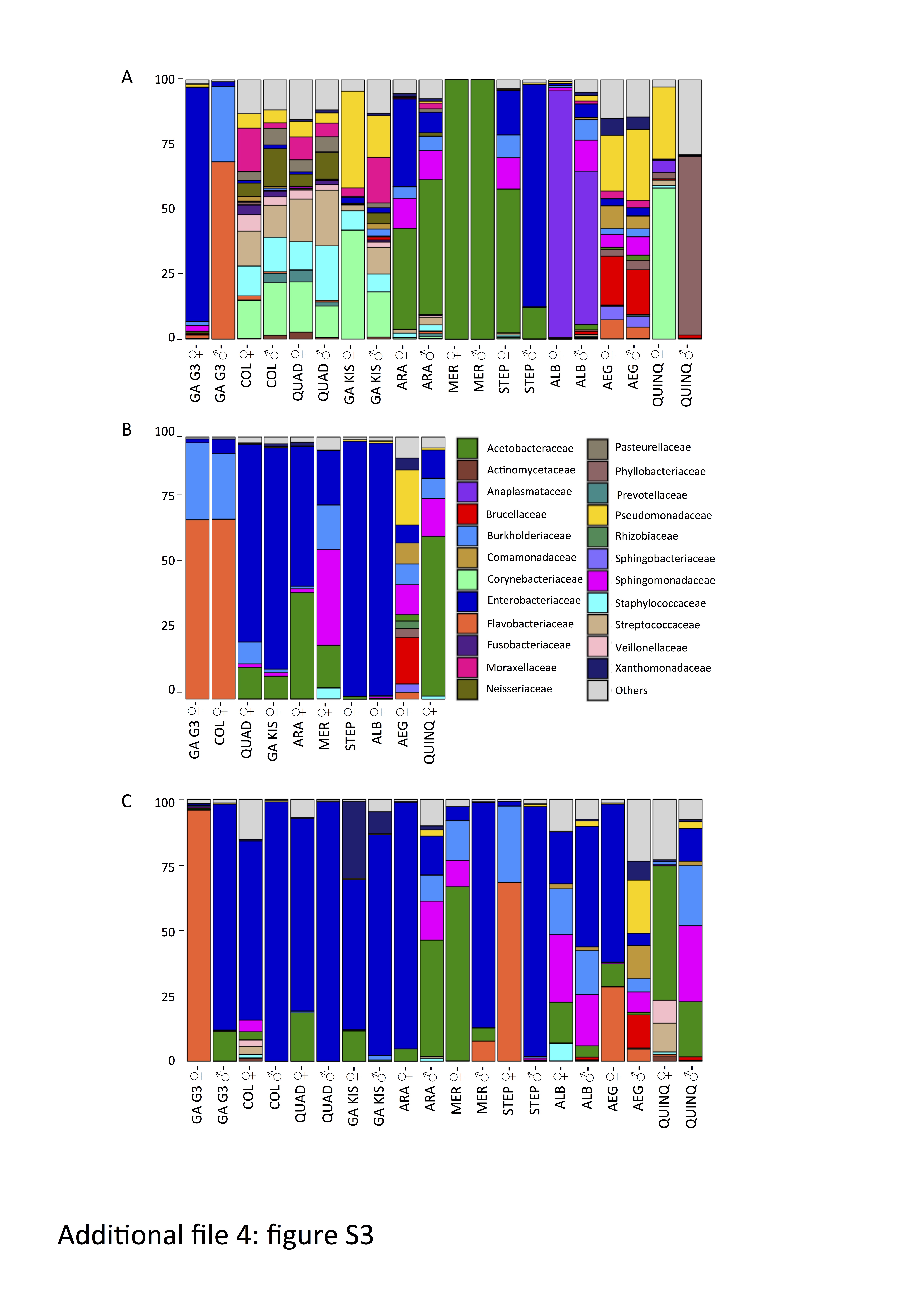

Supplement: Supplementary file 4 — Figure S3. Family level composition (% of OTUs) in different organs of nine mosquito species. Only OTUs representing > 1% of the total reads are represented here. The family color code is given for (A), (B) and (C). GA G3: An. gambiae G3; COL: An. coluzzii; QUAD: An. quadriannulatus; GA KIS: An. gambiae Kisumu; ARA: An. arabiensis; MER: An. merus; STEP: An. stephensi; ALB: Ae. albopictus; AEG: Ae. aegypti; QUINQ: Cx. quinquefasciatus; ♀: females; ♂: males. (JPG 3132 kb) [file 12866_2018_1266_MOESM4_ESM.jpg]
