# Supplementary material for: Estimating bacteria diversity in different organs of nine species of mosquito by next generation sequencing
Source: BMC Microbiol. 2018 Oct 4;18:126. doi: 10.1186/s12866-018-1266-9 (PMC6172810; doi:10.1186/s12866-018-1266-9)
Supplement: Supplementary file 3 — Table S1. Percentage of phyla OTUs in the reproductive organs (A) salivary glands (B) and guts (C). (DOCX 47 kb) [file 12866_2018_1266_MOESM3_ESM.docx]

**Table S1:** percentage of phyla OTUs in the reproductive organs (A) salivary glands (B) and guts (C).

**A:** Reproductive organs

| Phylum | ARA ♀ | ARA ♂ | AEG ♀ | AEG ♂ | ALB ♀ | ALB ♂ | QUINQ ♀ | QUINQ ♂ | QUAD ♀ | QUAD ♂ | COL ♀ | COL ♂ | GA KIS ♀ | GA KIS ♂ | GA G3  ♀ | GA G3  ♂ | STEP ♀ | STEP ♂ | MER  ♀ | MER ♂ |
| --- | --- | --- | --- | --- | --- | --- | --- | --- | --- | --- | --- | --- | --- | --- | --- | --- | --- | --- | --- | --- |
| Acidobacteria | 0 | 0 | 0 | 0 | 0 | 0 | 0 | 0 | 0 | 0 | 0 | 0,3 | 0 | 0 | 0 | 0 | 0 | 0 | 0 | 0 |
| Actinobacteria | 1,2 | 2,8 | 1,2 | 2,4 | 0,2 | 1,5 | 58,2 | 0,1 | 25,9 | 15,6 | 18 | 23,7 | 44,4 | 20,3 | 0,7 | 0 | 1,3 | 0 | 0 | 0 |
| Bacteroidetes | 0,6 | 3,6 | 14 | 10 | 0,1 | 0,5 | 0,1 | 0 | 5 | 3,3 | 3 | 6,2 | 0 | 1,6 | 1,5 | 68,4 | 0,4 | 0 | 0 | 0 |
| Chlamydiae | 0 | 0,1 | 0 | 0 | 0 | 0,1 | 0 | 0 | 0 | 0 | 0 | 0 | 0 | 0 | 0 | 0 | 0,1 | 0 | 0 | 0 |
| Cyanobacteria | 0,1 | 0,1 | 0 | 0 | 0 | 0,1 | 0,2 | 0 | 0 | 0 | 2,9 | 0,1 | 0 | 1,4 | 0 | 0 | 0 | 0 | 0 | 0 |
| Deinococcus-Thermus | 0 | 0 | 0 | 0 | 0 | 0 | 0 | 0 | 0 | 0 | 0,5 | 0 | 0 | 0,2 | 0 | 0 | 0 | 0 | 0 | 0 |
| Elusimicrobia | 0 | 0 | 0 | 0 | 0 | 0,2 | 0 | 0 | 0 | 0 | 0 | 0 | 0 | 0 | 0 | 0 | 0,1 | 0 | 0 | 0 |
| Firmicutes | 3,8 | 6,9 | 0,3 | 0,9 | 0 | 1,4 | 3,1 | 0,4 | 35,5 | 49 | 33,6 | 33,9 | 10,9 | 22 | 0,4 | 0 | 1,7 | 0 | 0 | 0 |
| Fusobacteria | 0 | 0,4 | 0,1 | 0 | 0 | 0 | 0 | 0 | 0,5 | 2,7 | 3,9 | 2,1 | 0,2 | 1,2 | 0 | 0 | 0,1 | 0 | 0 | 0 |
| Planctomycetes | 0 | 0 | 0 | 0 | 0 | 0 | 0 | 0 | 0 | 0 | 0 | 0,5 | 0 | 0 | 0 | 0 | 0 | 0 | 0 | 0 |
| Proteobacteria | 94,2 | 86,1 | 83,4 | 85,2 | 99,7 | 95,9 | 38,4 | 99,5 | 32,1 | 28,1 | 37,6 | 32,6 | 44,4 | 52,8 | 97,3 | 31,5 | 96,3 | 100 | 100 | 100 |
| SHA-109 | 0 | 0 | 0,9 | 1,4 | 0 | 0,2 | 0 | 0 | 0 | 0 | 0 | 0 | 0 | 0 | 0 | 0 | 0 | 0 | 0 | 0 |
| Other | 0 | 0 | 0 | 0 | 0 | 0 | 0 | 0 | 0 | 0,6 | 0 | 0 | 0 | 0 | 0 | 0 | 0 | 0 | 0 | 0 |
| Unassigned | 0 | 0,1 | 0 | 0 | 0 | 0 | 0 | 0 | 1 | 0,7 | 0,5 | 0,8 | 0 | 0,5 |  | 0,1 | 0 | 0 | 0 | 0 |

**B:** Salivary glands

| Phylum | ARA ♀ | AEG ♀ | ALB ♀ | QUINQ ♀ | QUAD ♀ | COL ♀ | GA KIS  ♀ | GA G3 ♀ | STEP ♀ | MER ♀ |
| --- | --- | --- | --- | --- | --- | --- | --- | --- | --- | --- |
| Acidobacteria | 0 | 0 | 0 | 0,2 | 0 | 0 | 0 | 0 | 0 | 0,1 |
| Actinobacteria | 0,2 | 0,3 | 0,8 | 0,8 | 0,1 | 0 | 0,1 | 0 | 0 | 1 |
| Bacteroidetes | 0,1 | 6,2 | 0 | 0,3 | 0 | 68,6 | 0,1 | 68,5 | 0 | 0,1 |
| Chlamydiae | 0 | 0 | 0 | 0 | 0 | 0 | 0 | 0 | 0 | 0,1 |
| Cyanobacteria | 0 | 0 | 0 | 0 | 0 | 0 | 0 | 0 | 0 | 0 |
| Deinococcus-Thermus | 0 | 0 | 0 | 0 | 0 | 0 | 0 | 0 | 0 | 0 |
| Elusimicrobia | 0 | 0,1 | 0 | 0,1 | 0 | 0 | 0 | 0 | 0 | 0 |
| Firmicutes | 0,5 | 0,2 | 0,1 | 2,2 | 0,2 | 0,2 | 0,2 | 0 | 0,1 | 5,2 |
| Fusobacteria | 0 | 0 | 0 | 0,1 | 0 | 0 | 0 | 0 | 0 | 0 |
| Planctomycetes | 0 | 0 | 0 | 0 | 0 | 0 | 0 | 0 | 0 | 0 |
| Proteobacteria | 99,2 | 92,6 | 99,1 | 96,2 | 99,7 | 31 | 99,6 | 31,4 | 99,9 | 93,5 |
| SHA-109 | 0,2 | 0 | 0 | 0 | 0 | 0 | 1,4 | 0 | 0 | 0,9 |
| Other | 0 | 0 | 0,6 | 0 | 0 | 0 | 0 | 0 | 0 | 0 |
| Unassigned | 0 | 0 | 0,7 | 1 | 0 | 0,1 | 0 | 0 | 0 | 0 |

**C**: Guts

| Phylum | ARA ♀ | ARA ♂ | AEG ♀ | AEG ♂ | ALB ♀ | ALB ♂ | QUINQ ♀ | QUINQ ♂ | QUAD ♀ | QUAD ♂ | COL ♀ | COL ♂ | GA KIS ♀ | GA KIS ♂ | GA G3  ♀ | GA G3  ♂ | STEP ♀ | STEP ♂ | MER  ♀ | MER ♂ |
| --- | --- | --- | --- | --- | --- | --- | --- | --- | --- | --- | --- | --- | --- | --- | --- | --- | --- | --- | --- | --- |
| Acidobacteria | 0 | 0 | 0 | 0,7 | 0 | 0 | 0 | 0 | 0 | 0 | 0 | 0 | 0 | 0 | 0 | 0 | 0 | 0 | 0 | 0 |
| Actinobacteria | 0 | 2,1 | 2,4 | 2,1 | 1,7 | 1,6 | 9 | 3,6 | 0 | 0,3 | 0 | 0,1 | 0 | 0 | 0,1 | 0 | 1,1 | 0 | 0 | 0 |
| Bacteroidetes | 0 | 0,8 | 9,1 | 0,4 | 0,6 | 0,7 | 4,8 | 1,5 | 0 | 0,1 | 0 | 95,9 | 0 | 28,5 | 0 | 68,4 | 0 | 0 | 7,8 | 0 |
| Chlamydiae | 0 | 0 | 0,1 | 0,5 | 0,2 | 0,2 | 0 | 0 | 0 | 0,1 | 0 | 0 | 0 | 0 | 0 | 0 | 0 | 0 | 0 | 0 |
| Cyanobacteria | 0 | 0,2 | 0 | 0,3 | 0 | 0 | 0,1 | 0 | 0 | 0 | 0 | 0 | 0 | 0 | 0 | 0 | 0 | 0 | 0 | 0 |
| Deinococcus-Thermus | 0 | 0 | 0 | 0,4 | 0 | 0 | 0 | 0 | 0 | 0 | 0 | 0 | 0 | 0 | 0 | 0 | 0 | 0 | 0 | 0 |
| Elusimicrobia | 0 | 0,1 | 0 | 0 | 0,2 | 0,2 | 0 | 0 | 0 | 0,1 | 0 | 0 | 0 | 0 | 0 | 0 | 0 | 0 | 0 | 0 |
| Firmicutes | 0 | 2 | 0,8 | 7,7 | 0,4 | 0,3 | 23,5 | 10,4 | 0 | 0,8 | 0 | 0,4 | 0 | 0 | 0 | 0 | 0 | 0 | 0 | 0,1 |
| Fusobacteria | 0 | 0 | 0,1 | 0 | 0 | 0 | 2,7 | 1,6 | 0 | 0 | 0 | 0 | 0 | 0 | 0 | 0 | 0 | 0 | 0 | 0 |
| Planctomycetes | 0 | 0 | 0 | 0 | 0,1 | 0,2 | 0 | 0 | 0 | 0,1 | 0 | 0 | 0 | 0 | 0 | 0 | 0 | 0 | 0 | 0 |
| Proteobacteria | 100 | 94,7 | 86,9 | 87,3 | 96,7 | 96,2 | 59,8 | 82,9 | 100 | 98,5 | 100 | 3,4 | 99,9 | 70,8 | 99,9 | 31,5 | 98,9 | 100 | 92 | 99,8 |
| SHA-109 | 0 | 0 | 0,5 | 0,4 | 0 | 0,5 | 0 | 0 | 0 | 0 | 0 | 0 | 0 | 0 | 0 | 0 | 0 | 0 | 0 | 0 |
| Other | 0 | 0 | 0 | 0 | 0 | 0 | 0 | 0 | 0 | 0 | 0 | 0 | 0 | 0 | 0 | 0 | 0 | 0 | 0 | 0 |
| Unassigned | 0 | 0 | 0 | 0 | 0 | 0,1 | 0,1 | 0 | 0 | 0 | 0 | 0,2 | 0 | 0,7 | 0 | 0 | 0 | 0 | 0,1 | 0 |

**Additional file 3: Table S1**
